# Virulence Factors of *Erwinia amylovora*: A Review

**DOI:** 10.3390/ijms160612836

**Published:** 2015-06-05

**Authors:** Núria Piqué, David Miñana-Galbis, Susana Merino, Juan M. Tomás

**Affiliations:** 1 Departament de Microbiologia i Parasiologia Sanitàries, Facultat de Farmàcia, Universitat de Barcelona, Av. Joan XXIII s/n, 08028 Barcelona, Spain; E-Mail: davidminyana@ub.edu; 2 Departament de Microbiologia, Facultat de Biologia, Universitat de Barcelona, Av. Diagonal 643, 08071 Barcelona, Spain; E-Mails: smerino@ub.edu (S.M.); jtomas@ub.edu (J.M.T.)

**Keywords:** *Erwinia amylovora*, virulence factors, plant pathogenesis, fire blight, type III secretion system, exopolysaccharide, amylovoran, biofilms, motility, quorum sensing

## Abstract

*Erwinia amylovora*, a Gram negative bacteria of the *Enterobacteriaceae* family, is the causal agent of fire blight, a devastating plant disease affecting a wide range of host species within *Rosaceae* and a major global threat to commercial apple and pear production. Among the limited number of control options currently available, prophylactic application of antibiotics during the bloom period appears the most effective. Pathogen cells enter plants through the nectarthodes of flowers and other natural openings, such as wounds, and are capable of rapid movement within plants and the establishment of systemic infections. Many virulence determinants of *E. amylovora* have been characterized, including the Type III secretion system (T3SS), the exopolysaccharide (EPS) amylovoran, biofilm formation, and motility. To successfully establish an infection, *E. amylovora* uses a complex regulatory network to sense the relevant environmental signals and coordinate the expression of early and late stage virulence factors involving two component signal transduction systems, bis-(3′-5′)-cyclic di-GMP (c-di-GMP) and quorum sensing. The LPS biosynthetic gene cluster is one of the relatively few genetic differences observed between *Rubus*- and *Spiraeoideae*-infecting genotypes of *E. amylovora*. Other differential factors, such as the presence and composition of an integrative conjugative element associated with the Hrp T3SS (*hrp* genes encoding the T3SS apparatus), have been recently described. In the present review, we present the recent findings on virulence factors research, focusing on their role in bacterial pathogenesis and indicating other virulence factors that deserve future research to characterize them.

## 1. Introduction

*Erwinia amylovora* is the type species of the genus *Erwinia* that belongs to the family *Enterobacteriaceae*. As is common in bacterial taxonomy in recent years, there have been a continuous description of novel *Erwinia* species as well as some species have been reclassified and transferred to other genera (http://www.bacterio.net). Most members of this genus cause diseases in plants and, historically, it is important to remember that *E. amylovora* was the first bacterium demonstrated to cause disease in plant, a discovery made in the late 1800s, at the same period as a similar discovery with human and animal diseases [1,2].

*E. amylovora* causes fire blight, a devastating plant disease affecting a wide range of host species within the *Rosaceae* subfamily Spiraeoideae, and is a major global threat to commercial apple and pear production. Moreover strains infecting plants in the genus *Rubus* belonging to the subfamily Rosoideae, including blackberry and raspberry, have also been reported [3,4,5,6,7].

In the last two centuries this pathogen has spread worldwide [6,7,8] and, in consequence, *E. amylovora* has been cataloged as a quarantine organism in the European Union, where it is subject to phytosanitary legislation [8], and, recently, has been included in the top 10 plant pathogenic bacteria published in the journal *Molecular Plant Pathology* [9].

Among the limited number of control options currently available, prophylactic application of antibiotics (e.g., streptomycin or oxytetracycline) during the bloom period appears most effective [10]. However, regulatory restriction, public health concerns, and pathogen resistance development severely limit the long-term prospects of antibiotic use [11,12]. Biological control measures may offer promising alternatives to minimize or even substitute the use of antibiotics, and to mitigate occurrence of resistance [5,13].

In the pathogenesis of *E. amylovora*, pathogen cells enter plants through the nectarthodes of flowers and other natural openings, such as wounds, and are capable of rapid movement within plants and the establishment of systemic infections [14,15,16,17]. In susceptible hosts, bacteria first move through the intercellular spaces of parenchyma and, in a later stage, in the xylem vessels, thus provoking extensive lesions, and sometimes complete dieback of the tree, under favorable climatic conditions. The diseased parts of the plant become brown or black, as if they had been swept by fire [17].

Many virulence determinants of *E. amylovora* have been characterized, including the Type III secretion system (T3SS), the exopolysaccharide (EPS) amylovoran, biofilm formation, and motility [15,18]. To successfully establish an infection, *E. amylovora* uses a complex regulatory network to sense the relevant environmental signals and coordinate the expression of early and late stage virulence factors involving two component signal transduction systems, bis-(3′-5′)-cyclic di-GMP (c-di-GMP) and quorum sensing [15]. In the present review, we present the recent findings on virulence factors of *E. amylovora* research, focusing on their role in bacterial pathogenesis and on the aspects that deserve future research.

## 2. Virulence Factors

*E. amylovora* is highly virulent and capable of rapid systemic movement within plant hosts and of rapid dissemination among rosaceous species, including apple and pear trees, when environmental conditions are favorable. The internal movement of the pathogen through the vascular system of plants and the ability of the pathogen to infect flowers, actively growing shoots, and rootstocks makes the management of fire blight difficult [14,19].

It has been shown that two major virulence determinants are required for *E. amylovora* to infect and cause disease on host plants: the EPS amylovoran and the Hrp type III secretion system (T3SS) [20]. Previous results demonstrated that *E. amylovora* forms a biofilm *in vitro* and *in planta* [19,21,22].

### 2.1. Exopolysaccharides (EPS) Amylovoran and Levan

Exopolysaccharides (EPS) have been suggested to play a key role in bypassing the plant defense system, in disturbing and obstructing the vascular system of the plant and in protecting the bacteria against water and nutrient loss during dry conditions [19,23,24,25]. Previous work has demonstrated that EPS is an important element in the biofilm formation of *E. amylovora*, enabling the bacteria to attach to several surfaces and to each other [19,21,26]. Amylovoran has been shown to be the main factor necessary for biofilm formation, and levan is a contributing factor [21,24], the quantity of amylovoran produced by individual *E. amylovora* strains being correlated with the degree of virulence [24,27].

Amylovoran is a polymer of a pentasaccharide repeating unit that generally consists of four galactose residues and one glucuronic acid residue [24,27,28]. The molecular size of amylovoran is influenced by several environmental conditions and cell-metabolism-related factors [24,29]. The strains of *E. amylovora* that do not have the capacity to produce amylovoran are non-pathogenic and are unable to spread in plant vessels [24,30].

The amylovoran synthesis (*ams*) gene cluster involved in the biosynthesis of amylovoran produces 12 *ams*-encoded gene products (AmsA to AmsL). AmsC, AmsH, and AmsL are believed to be involved in oligosaccharide transport and assembly, while AmsA possesses a tyorisine kinase activity. AmsB, AmsD, AmsE, AmsG, AmsJ, and AmsK proteins appear to play a part in annealing the different galactose, glucuronic acid, and pyruvyl subunits to the lipid carrier in order to form an amylovoran unit. AmsF may process newly synthesized repeating units and/or be involved in their polymerization by adding them to an existing amylovoran chain. Finally, AmsI seems to have a distinct function in recycling of the diphosphorylated lipid carrier after release of the synthesized repeating unit [24,31,32,33].

Levan is another EPS produced by *E. amylovora*, considered as a virulence factor [19,34]. It has been shown that the lack of levan synthesis can result in a slow development of symptoms in the host plant [24,35]. The specific role of levan in pathogenesis is still unknown and deserves further research.

Interestingly, in recent studies, it has been demonstrated that the depolymerase (DpoL1) encoded by the T7-like *E. amylovora* phage L1 efficiently degrades amylovoran. Exposure of the bacteria to either L1 phage or recombinant DpoL1 led to EPS degradation and a phenotype similar to the one observed with EPS mutants. The enzyme strips the cells, thereby paving the way for infection by providing access of L1 phage to the cell wall, and also facilitating the infection by other phages, as Dpo-negative Y2 phages [5].

### 2.2. Type III Secretion System (T3SS)

**Figure 1:**
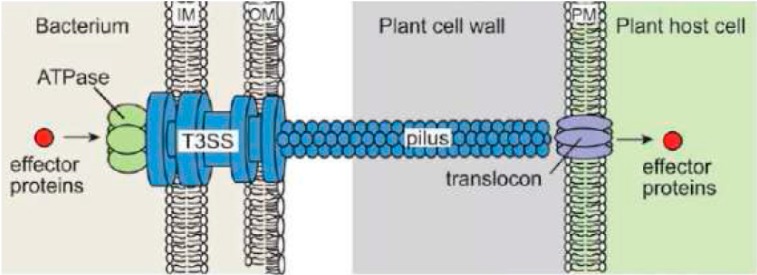
Schematic representation of the T3SS from plant pathogenic bacteria [39] (modified from [39], with permission from American Society of Plant Biologists).

The type III secretion system (T3SS) is one of the important virulence factors used by *E. amylovora* in order to successfully infect its hosts [24,36]. As with other Gram negative phytopathogenic bacteria, *E. amylovora* uses this evolutionarily conserved secretion system to export and deliver effector proteins into the cytosol of host plant cells through a pilus-like structure, which forms the central core element of T3SS [24] (Figure 1).

*E. amylovora* pathogenicity relies on a type III secretion system and on a single effector DspA/E. This effector belongs to the widespread AvrE family of effectors whose biological function is not fully understood [37].

T3SS is composed of a large, cylindrically shaped macromolecular complex organized into a series of ring-like structures with inner rings, outer rings and neck structure. It is embedded in the inner and outer membrane of the bacteria, while spanning the periplasmic membrane and extending into the extracellular environment with a pilus filament [24,37] (Figure 1). It has been shown that T3SS occurs only at the site of Hrp pilus assembly and that pilus guides the transfer of effector proteins outside the bacterial cell, favoring the “conduit/guiding filament” model [37] (Figure 1).

The T3SS of plant-pathogenic bacteria is mainly made out of Hrc proteins, encoded by *hrp*-conserved (*hrc*) genes among plant-pathogenic bacteria and Hrp proteins, encoded by hypersensitive response and pathogenicity (*hrp*) genes. In *E. amylovora*, *hrc* and *hrp* genes are clustered in a pathogenicity island, which contains four regions, *i.e.*, an *hrc*/*hrp* region, an Hrc effectors and elicitors region, an Hrp-associated enzymes region, and an island transfer region [24,38].

### 2.3. Curli

Curli are the major proteinaceous component of a complex extra-cellular matrix produced by many *Enterobacteriaceae*, including *E. amylovora*. Curli fibres are known to be involved in adhesion to surfaces, cell aggregation and biofilm formation. Curli can also mediate host cell adhesion and invasion and they stimulate the host inflammatory responses [19,40,41]. The structure and biogenesis of curli are unique among the bacterial fibres that have been described to date, belonging to a growing class of fibres, known as amyloids, which are associated with diverse neurological diseases in humans. Curli fibres are 4–6 nm-wide and, like other amyloid fibres, are β sheet-rich self-assembling protein polymers that are resistant to chemical and temperature denaturation, and to digestion by proteinases [41].

Recent results have showed reductions in virulence due to deletion of a regulator of curli genes, *crl*, suggesting that not only functional attachment but also mature biofilm formation is needed for full virulence in the host [19]. Further research is necessary to charaterize the chemical structures and genes encoding this structure and its specific role in pathogenicity, as it is also in process for other Enterobacteria as *E. coli* or *Salmonella* [40].

### 2.4. Biofilm Formation

In addition to the specific role of the exopolysaccharides amylovoran and levanin in the biofilm formation of *E. amylovora*, as mentioned above, a recent study has suggested that type I fimbriae, flagella, type IV pili, and curli of *E. amylovora* may contribute to biofilm formation in static and flowing environments and that defects in many of the genes encoding these appendages result in decreased virulence in planta [19].

Previous results of the same group demonstrated that *E. amylovora* forms a biofilm *in vitro* and *in planta* [19,21]. Pathogenesis and biofilm formation appear to be linked, but without identifying genes encoding traits independent of amylovoran production, the mechanistic role in virulence of biofilm formation in *E. amylovora* could not be studied. Interestingly, mutants with reduced biofilm-formation ability appear unable to successfully establish large populations in apple xylem. Colonization of xylem is critical to the systemic movement of the pathogen through plants [19,22]. Therefore, biofilm-deficient mutants remain localized within an inoculated leaf and are strongly impaired in the ability to invade the rest of the plant [19].

**Figure 2:**
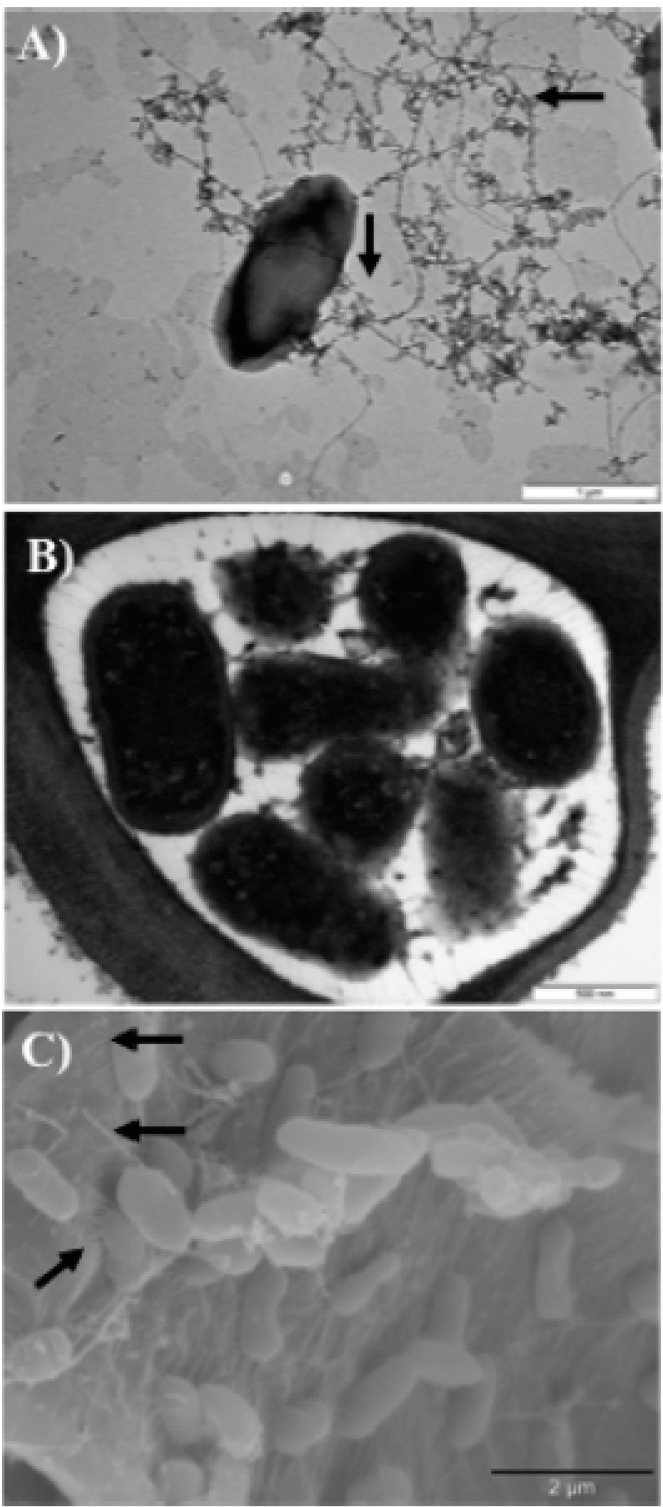
Images of putative attachment structures of *E. amylovora* [19] (adapted from [19], with permission from American Society for Microbiology). (**A**) TEM imaging of a planktonic *E. amylovora* cell grown in broth culture and negatively stained. Peritrichous flagella are indicated by arrows (scale: 1 µm); (**B**) TEM image of *E. amylovora* in planta. Putative attachment structures connect bacterial cells to host cells (scale: 1 µm); (**C**) SEM image of *E. amylovora* cells found within a biofilm, with multiple appendages that protrude from the bacterial cell and attach to the host surface, as indicated by the arrows (scale: 2 µm).

In a study using a bioinformatic approach and the recently sequenced genome of *E. amylovora* [19,42,43], genes encoding putative cell surface attachment structures were identified [19]. A time course assay indicated that type I fimbriae function earlier in attachment, while type IV pilus structures appeared to function later in attachment. Deficiencies in type I fimbriae lead to an overall reduction in *E. amylovora* virulence. By deletion of individual genes and gene clusters and using a combination of *in vitro* attachment assays and plant virulence assays it was demonstrated that multiple attachment structures are present in *E. amylovora* and play a role in mature biofilm formation, which is critical to pathogenesis and systemic movement in the host (Figure 2). In contrast, a fully functional biofilm was not necessary to survival and growth *in planta* [19].

Although the mechanistic details behind biofilm formation remain largely unknown and genetic and structural analyses should be done for full characterization of these structures, it is suggested that they are formed in response to environmental triggers [24,44] and quorum sensing signals [24,45].

### 2.5. Motility

*E. amylovora* is a nonobligate pathogen able to survive outside the host under starvation conditions, allowing its spread by various means, such as rainwater [46]. In fact, motility mechanisms have been shown to be of relevance for the survival of the bacteria ouside the host [46] and also for the attachment to the host cell surfaces, together with other virulence factors as type IV pili, type I fimbriae and curli by biofilm formation [19]. In different studies, long peritrichous flagella have been observed (Figure 2) [19,46], although chemical structural data have not been published yet.

In a study where two of the four gene clusters encoding the production and regulation of flagella in *E. amylovora* were deleted, various effects of flagella on biofilm formation and virulence were demonstrated. Results obtained also showed that flagellum production appeared to be controlled by multiple gene clusters that may function independently. Consequently, it was suggested that the flagella of *E. amylovora* have multifaceted functions in the biofilm formation process [19].

Swarming motility has also been described in *E. amylovora* [15,47], although, as in other *Enterobacteria*, its role remains unknown, requiring further research.

*E. amylovora* responses to starvation revealed that cells lost motility but most of them exhibited long peritrichous flagella attached to the bacterial surface. Motility inactivation during starvation would be reversible since flagella could enable a rapid recovery of motility under favorable conditions [46].

### 2.6. Lipopolysaccharide (LPS)

LPS is a factor that has been described recently to be involved in the virulence of *E. amylovora* [48], although the complete chemical structure of LPS and its relationship with the pathogenicity has not yet been described.

Interestingly, in *Erwinia carotovora*, a comprehensive physicochemical analysis of highly purified mono- and bis-phosphoryl hexa- to heptaachyl and pentaacyl lipid A part structures has been published. The distinct differences observed between different Lipid A structures could explain differences in the biological activity, such as the cytokine inducing activity [49].

At the genetic level, comparative genomic analysis revealed differences in the LPS biosynthesis gene cluster between the *Rubus*-infecting strain ATCC BAA-2158 and the Spiraeoideae-infecting strain CFBP 1430 of *E. amylovora*. These differences were restricted to the core region that could be involved in a process of adaptation to the new host. Genetic differences observed in the LPS biosynthetic gene cluster corroborate *rpoB*-based phylogenetic clustering of *E. amylovora* into four different groups and enable the discrimination of Spiraeoideae- and *Rubus*-infecting strains [48].

### 2.7. Metalloprotease PrtA

A metalloprotease with a molecular mass of 48 kDa secreted by *E. amylovora* has been characterized [50]. The protease is apparently secreted into the external medium through the type I secretion pathway via PrtD, PrtE, and PrtF, which share more than 90% identity with the secretion apparatus for lipase of *S. marcescens* [50].

A gene cluster encodes four genes connected to protease expression, including a structural gene (*prtA*) and three genes (*prtD*, *prtE*, *prtF*) for secretion of the protease, which are transcribed in the same direction. The organization of the protease gene cluster in *E. amylovora* is different from that in other Gram-negative bacteria, such as *Erwinia chrysanthemi*, *Pseudomonas aeruginosa*, and *Serratia marcescens*. On the basis of the conservative motif of metalloproteases, PrtA has been identified to be a member of the metzincin subfamily of zinc-binding metalloproteases, and has been confirmed to be the 48 kDa protease on gels by sequencing of tryptic peptide fragments derived from the protein [50]. In a protease mutant created by mutations with Tn 5–insertions in the *prtD* gene, the lack of protease reduced colonization of an *E. amylovora* secretion mutant, labeled with the gene for the green fluorescent protein (*gfp*) in the parenchyma of apple leaves [50].

Further research is necessary, however, to assess its structural characteristics, activity, substrates, the specific role of this metalloprotease in the pathogenic mechanisms of *E. amylovora*, and also the possible practical applications of this protein, based on the differences observed with other Gram negative bacteria, as already proposed for this interesting wide family of proteins [51].

### 2.8. Iron-Scavenging Siderophore Desferrioxamine

Under iron-limiting conditions, *E. amylovora* CFBP 1430 produces hydroxamate-type siderophores [52], characterized as cyclic desferrioxamines (DFOs), mostly DFO E [53,54] and the specific receptor FoxR, a 70,000-Da protein needed for the passage of ferric complexes across the outer membrane [17,54].

Several iron uptake negative mutants of *E. amylovora* CFBP 1430 isolated by insertional mutagenesis have been identified. In mutant VD61, the mutation (dfo-61::MudIIpR13) disrupts the DFO biosynthetic pathway; in VD17, the mutation (foxR-17::MudIIpR13) affects the synthesis of the FoxR receptor and the mutant accumulates DFO in the external medium because of its failure to transport back the DFO ferric complex [17,54]. In the pathogenic analysis of these mutants, results obtained demonstrated that, in *E. amylovora*, production of DFOs is a critical function for bacterial iron acquisition during pathogenesis, as well as for bacterial induction of electrolyte leakage from plant cells [17]. Based on this, a dual role of DFO during plant/*E. amylovora* interactions has been proposed [17], which deserve further research.

### 2.9. Multidrug Efflux Pump AcrAB

During pathogenesis, *E. amylovora* is exposed to a variety of plant-borne antimicrobial compounds, which in the case of plants of *Rosaceae*, many are constitutively synthesized as isoflavonoids [22]. Then, bacterial multidrug efflux transporters, which mediate resistance toward structurally-unrelated compounds, might confer tolerance to these antimicrobial compounds (phytoalexins) [22]. In this regard, it has been observed that clonation of the *acrAB* locus from *E. amylovora*, encoding a resistance nodulation division-type transport system, in *E. coli* conferred resistance to hydrophobic and amphiphilic toxins [22]. An *acrB*-deficient *E. amylovora* mutant was impaired in virulence on apple rootstock MM 106 and was susceptible toward extracts of leaves of MM 106, as well as to the apple phytoalexins phloretin, naringenin, quercetin, and (+)-catechin [22]. These results strongly suggest that the AcrAB transport system plays an important role as a protein complex required for the virulence of *E. amylovora* in resistance toward apple phytoalexins and that it is required for successful colonization of a host plant [22].

### 2.10. Other Virulence Factors

Other virulence factors of *E. amylovora* have been proposed, as the case of the sorbitol transporter, encoded by the *srl* operon [55]. It has been shown that expression of the *srl* operon in *E. amylovora* is high in the presence of sorbitol in medium and is repressed by glucose. Mutants with a sorbitol deficiency were still virulent on slices of immature pears, but were unable to cause significant fire blight symptoms on apple shoots. Since sorbitol is used for carbohydrate transport in host plants of *E. amylovora*, this sugar alcohol has been proposed to be an important factor in determining host specificity for the fire blight pathogen [55].

Moreover, Rosaceous plants also contain sucrose as storage and transport carbohydrates [56,57,58]. The regulation and biochemistry of sucrose metabolism of *E. amylovora* has also been studied, demonstrating that sucrose mutants (in the *scr* regulon), created by site-directed mutagenesis, did not produce significant fire blight symptoms on apple seedlings, thus, indicating the importance of sucrose metabolism for colonization of host plants by *E. amylovora* [58].

## 3. Genetics

The genome of *E. amylovora* consists of a circular chromosome of 3,805,874 bp and two plasmids: AMYP1 (28,243 bp), also reported as PEA29, and the larger plasmid AMYP2 (71,487 bp), also named pEA72. The small size of the *E. amylovora* genome (3.8 Mb) in comparison with most free-living enterobacteria, including plant pathogens, with genomes of 4.5–5.5 Mb, and the preponderance of pseudogenes, genome reduction can have occurred via mutational inactivation and subsequent deletion and, as a consequence, has led to the loss of most of the genes involved in anaerobic respiration and fermentation found in typical, related enterobacteria, with the consecuent reduction of the capacity to live in anaerobic environments [42]. The genome sequence of *E. amylovora* has revealed clear signs of pathoadaptation to the rosaceous plant environment. For example, T3SS-related proteins are more similar to proteins of other plant pathogens than to proteins of closely related enterobacteria [42].

Comparative genomic analysis of 12 strains representing distinct populations (e.g., geographic, temporal, host origin) of *E. amylovora* has allowed to describe the pan-genome of this species. The pan-genome contains 5751 coding sequences and is a highly conserved relative to other phytopathogenic bacteria, comprising on average 89% conserved core genes. While the chromosones of Spiraeoideae-infecting strains are highly homogeneous, greater genetic diversity was observed between Spiraeoideae- and *Rubus*-infecting strains (and among individual *Rubus*-infecting strains) [20].

Using molecular approaches based on the study of repetitive elements, such as Multiple Loci Variable Number of Tandem Repeats Analysis (MLVA) [59] or sequencing of Clustered Regularly Interspaced Short Palindromic Repeats (CRISPR) [60], this diversity has also been especially observed among strains isolated from *Rubus* plants and, to a lesser extent, in Spiraeoideae-infecting strains [48].

In comparison with other plant pathogens, however, *E. amylovora* has relatively low genetic diversity, with a limited genetic recombination, this being associated with its exposure to limited selection pressures due to pome fruit breeding strategically favoring only high-valued varieties, which are often highly susceptible to fire blight [20,61,62].

On the basis of the current available data, it has been hypothesized that the critical event for adaptation to *Rubus* spp. took place after species separation of *E. amylovora* and *E. pyrifoliae*, as the Spiraeoideae infecting isolates of *E. amylovora* and *E. pyrifoliae* (including Japanese strains), as well as *E. tasmaniensis* and *E. piriflorinigrans*, all sharing the Spiraeoideae-type LPS biosynthetic cluster [48]. Interestingly, genome sequencing of three Mexican *E. amylovora* strains have revealed an *rpsL* chromosal mutation conferring high-level of streptomycin resistance [63]. This chromosomal mutation is the predominant *E. amylovora* mechanism, typically appearing after years of intensive application with long persistence in populations [10,63], explaining the observed inefficacy of streptomycin in Mexico [63,64] and underscoring the need of revision of pesticide use strategies to avoid similar resistance evolution against other antimicrobial drugs as oxytetracycline or gentamicin [63].

The expression of genes related to starvation, oxidative stress, motility, pathogenicity, and virulence have been detected during the entire experimental period with different regulation patterns observed during the first 24 h. Further, starved cells remained as virulent as nonstressed cells. Overall, these results provide new knowledge on the biology of *E. amylovora* under conditions prevailing in nature, which could contribute to a better understanding of the life cycle of this pathogen [46].

No major differences among 12 strains of *E. amylovora* were reported in the amylovoran biosynthesis cluster (>98% amino acid indentity across the whole region) [20]. Variation, however, has been observed in the Hrp cluster, a pathogenicity island that encodes the hypersensitive response and pathogenicity (hrp) T3SS and the majority of known T3SS effector proteins [20,38]. Variation was identified in HrpK, the putative chaperones OrfA and OrfC (which varied between host specific grouping of *Rubus*- and Spiraeoideae-infecting strains) and, more significantly, Eop 1, which has been shown to function as a host-limiting factor [20,38,65,66].

Three gene clusters related to the Type 6 Secretion System (T6SS) have also been identified in *E. amylovora* [20,43], although their exact role in this species is unknown [20] and should deserve further research. In other bacteria, T6SS has been shown to play a significant role in bacterial-bacterial and bacterial-host interactions, suggesting a role for niche specialization [67].

## 4. Regulation of Virulence Factors

### 4.1. Two Component Transduction Systems

The phoPQ two component transduction system has been genetically characterized in *E. amylovora* [68] and it has been demonstrated that this system in *E. amylovora* plays major roles in virulence on immature pear fruit and in regulating amylovoran biosynthesis and swarming motility [47]. It has also been shown that phoPQ mutants were more resistant to strong acidic conditions (pH 4.5 or 5) than that of the wild-type (WT) strain, suggesting that this system in *E. amylovora* may negatively regulate acid resistance gene expression. Furthermore, the PhoPQ system negatively regulated gene expression of two novel T3SS in *E. amylovora*. These results are in contrast to those reported for the PhoPQ system in *Salmonella* and *Xanthomonas*, where it positively regulates T3SS and acid resistance. In addition, survival of phoPQ mutants was about 10-fold lower than that of WT when treated with cecropin A at pH 5.5, suggesting that the PhoPQ system renders the pathogen more resistant to cecropin A [68].

These results suggest that the the PhoPQ system may act as a part of a regulatory network that governs *E. amylovora* in a wide variety of cellular functions, thus enhancing its survival under the changing environments of different hosts or in different environments, such as the acidic plant apoplast, a major barrier for plant pathogenic bacteria to cause disease [68].

### 4.2. Small RNA

Recently, different regulatory small RNAs (sRNAs) requiring the RNA chaperone Hfq for both stability and functional activation have been identified in *E. amylovora*, and further, being some of them involved in the regulation of various virulence traits including motility, amylovoran EPS production, biofilm formation, and T3SS. This discovery is of relevance since, although sRNAs have been increasingly recognized as pivotal regulators in bacteria, genome-wide identification of sRNAs has only been performed in a limited number of bacteria [15].

### 4.3. Quorum Sensing

Quorum sensing systems have been described in *E. amylovora*, both QS-1, relying on an *N*-homoserine lactone autoinducer-1 signal (AI-1) [69,70,71] and QS-2 reliant on LuxS as the enzyme responsible for signal (AI-2) production [72]. However, a global regulatory function has not yet been explored. Although previous studies reported that *luxS* did not affect quorum sensing in *E. amylovora* [71], recent results have indicated that some strains of *E. amylovora* have *luxS-*dependent AI-2 activity, and that inactivation of *luxS* affects some phenotypes, including virulence *in* *planta* [72], although the molecular basis of *luxS*-dependent regulation remains to be determined [72]. It was observed that AI-2 produced in *E. amylovora* activated the expression of bioluminescence in the *Vibrio harveyi* BB170 reporter strain. Findings showed that AI-2 in *Ea*1665 was dependent on *EaluxS* because expression of *EaluxS* in the *luxS* frame-shift mutation of *E. coli* DH5α demonstrated its importance in AI-2 synthesis. The pattern of AI-2 production with respect to growth phase and environmental changes deserves further research, and may give some insight into the function of this signaling system [72].

Moreover, using comparative genomic analyses to search homologs of genes involved, several genes were identified corresponding to the Autoinducer-3/Epinephrine/Norepinephrine signaling system in *E. coli*. Particularly, the putative homologs to the two component system QseEF and the transcriptional regulator QseA are highly conserved in *E. amylovora*. Gene-knockout experiments revealed that the QseEFEa system is involved in the regulation of swarming motility, biosynthesis of amylovoran, and biofilm formation [73].

### 4.4. c-di-GMP

It has been shown that *E. amylovora* encodes eight genes involved in the biosynthesis and/or degradation of cyclic-di-GMP (c-di-GMP), a second messenger signaling compound. Individual knockout mutations in each of these genes revealed that c-di-GMP positively regulated biofilm formation and negatively regulated motility. These results demonstrated that *E. amylovora* possesses several regulatory networks that govern the expression of virulence genes [73,74].

### 4.5. ppGpp

In a recent study, the role of the bacterial alarmone ppGpp in activating the T3SS and virulence of *E. amylovora* was investigated using ppGpp mutants generated by Red recombinase cloning. The virulence of a ppGpp-deficient mutant (ppGpp(0)), as well as a *dksA* mutant of *E. amylovora*, was completely impaired, and bacterial growth was significantly reduced, suggesting that ppGpp is required for full virulence of *E. amylovora*. Expression of T3SS genes was greatly downregulated in the ppGpp(0) and *dksA* mutants. Western blotting showed that accumulations of the HrpA protein in the ppGpp(0) and dksA mutants were about 10% and 4%, respectively, of that in the wild-type strain. Furthermore, higher levels of ppGpp resulted in a reduced cell size of *E. amylovora*. Moreover, serine hydroxamate and α-methylglucoside, which induce amino acid and carbon starvation, respectively, activated hrpA and hrpL promoter activities in hrp-inducing minimal medium. These results demonstrated that ppGpp and DksA play central roles in *E. amylovora* virulence and indicated that *E. amylovora* utilizes ppGpp as an internal messenger to sense environmental/nutritional stimuli for regulation of the T3SS and virulence [75].

### 4.6. Type 3 Effectors

There are several effectors in *E. amylovora* that have been described [36], although recent findings have been focused on DspA/E since it has been shown that the ability of *E. amylovora* to promote disease mainly depends on this single injected T3E [76]. DspA/E belongs to the widespread AvrE family of type III effectors that suppress plant defense responses and promote bacterial growth following infection [76,77,78]. In recent studies, expression of dspA/E in the yeast *Saccharomyces cerevisiae* inhibited cell growth, this effect associated with perturbations of the actin cytoskeleton and endocytosis [77], activation of phosphatase 2A, and downregulation of the sphingolipid biosynthetic pathway leading to growth arrest [76].

In nonhost *Arabidopsis thaliana* leaves, DspA/E was required for transient bacterial growth, as an *E. amylovora* *dspA/E* mutant was unable to grow. Study of transgenic *Arabidopsis* lines expressing DspA/E indicated that DspA/E promotes modifications of plant cell metabolism among which the repression of protein synthesis could be determinant in the facilitation of necrosis and bacterial growth [78].

### 4.7. Hrp X/Y, HrpS and HrpL Cascade Leading to Activation of hrpT3SS

Two regulatory components, *hrpX* and *hrpY*, of the *hrp* system of *Erwinia amylovora* have been identified [79]. The *hrpXY* operon is expressed in rich media, but its transcription is increased threefold by low pH, nutrient, and temperature levels, conditions that mimic the plant apoplast. *hrpXY* is autoregulated and directs the expression of *hrpL*, which, in turn, activates transcription of other loci in the *hrp* gene cluster [80].

The deduced amino-acid sequences of *hrpX* and *hrpY* are similar to bacterial two-component regulators including VsrA/VsrD of *Pseudomonas* (*Ralstonia*) *solanacearum*, DegS/DegU of *Bacillus subtilis*, and UhpB/UhpA and NarX/NarP, NarL of *Escherichia coli*. The N-terminal signal-input domain of HrpX contains PAS domain repeats [79].

*hrpS*, located downstream of hrpXY, encodes a protein with homology to WtsA (HrpS) of *Erwinia* (*Pantoea*) *stewartii*, HrpR and HrpS of *Pseudomonas syringae*, and other σ^54^-dependent, enhancer binding proteins. Transcription of *hrpS* also is induced under conditions that mimic the plant apoplast. However, *hrpS* is not autoregulated, and its expression is not affected by *hrpXY* [79]. As a member of the NtrC family, HrpS is unusual in that it lacks a long N-terminal receiver domain [79].

The structure of the input domain of *E. amylovora* HrpX appears to be exceptional, compared with sensor proteins involved in other type III systems, which contain two transmembrane regions and a periplasmic domain [79]. Thus, at least two types of transmitter-receiver systems appear to have evolved for control of type III systems in response to environmental stimuli in hosts [79].

### 4.8. Regulatory Cascade for Amylovoran Synthesis

The biosynthesis of amylovoran is regulated by another two-component signal transduction system, the RcsCDB phosphorelay system, which has been found to be essential for virulence in *E. amylovora* [47].

## 5. Discussion

Nowadays, there is a high amount of interest to find and apply biological measures to combat and prevent plant diseases to avoid the use of chemical compounds in agriculture [81,82]. In this context, it is fundamental to understand the host (plant)-parasite interactions [81].

In this review, we have summarized the most recent findings regarding virulence factors of *E. amylovora*, having seen that, although the most relevant virulence mechanisms have been described, there is substantial further research to completely characterize, at the chemical and functional levels, the different factors involved in pathogenicity. In this regard, recent genome sequencing should accelerate the knowledge on this species, particularly in regards to pathogenicity, considering the limited genetic diversity observed in the different strains. Overall, these data will help to understand the whole mechanism of the specific interactions of *E. amylovora* with its hosts, thus, allowing better diagnosis and control strategies, which can include biological measures, such as the design of lytic bacteriophages, competitive attenuated strains, *etc*.

For most of the virulence factors identified, even for the two main factors (the EPS amylovoran and T3SS), further research is necessary to fully understand their role in the pathogenic network of the bacteria, including the main mechanism of adhesion via the biofilm and the interaction of the different elements with the plant structures, including the acidic plant apoplast and the plant defenses, under different environmental conditions.

Complete characterization of the structures involved in virulence, and their regulation mechanisms and further genetic analysis, will allow to design more precise diagnostic and control strategies (including a more rational use of antimicrobials), together with a more precise phylogenetic classification of *E. amylovora*, across the genus, and the Enterobacterial family.
